# Supplementary figures and images for: Effects of glycosaminoglycan content in extracellular matrix of donor cartilage on the functional properties of osteochondral allografts evaluated by micro-CT non-destructive analysis
Source: PLoS One. 2023 May 23;18(5):e0285733. doi: 10.1371/journal.pone.0285733 (PMC10204946; doi:10.1371/journal.pone.0285733)

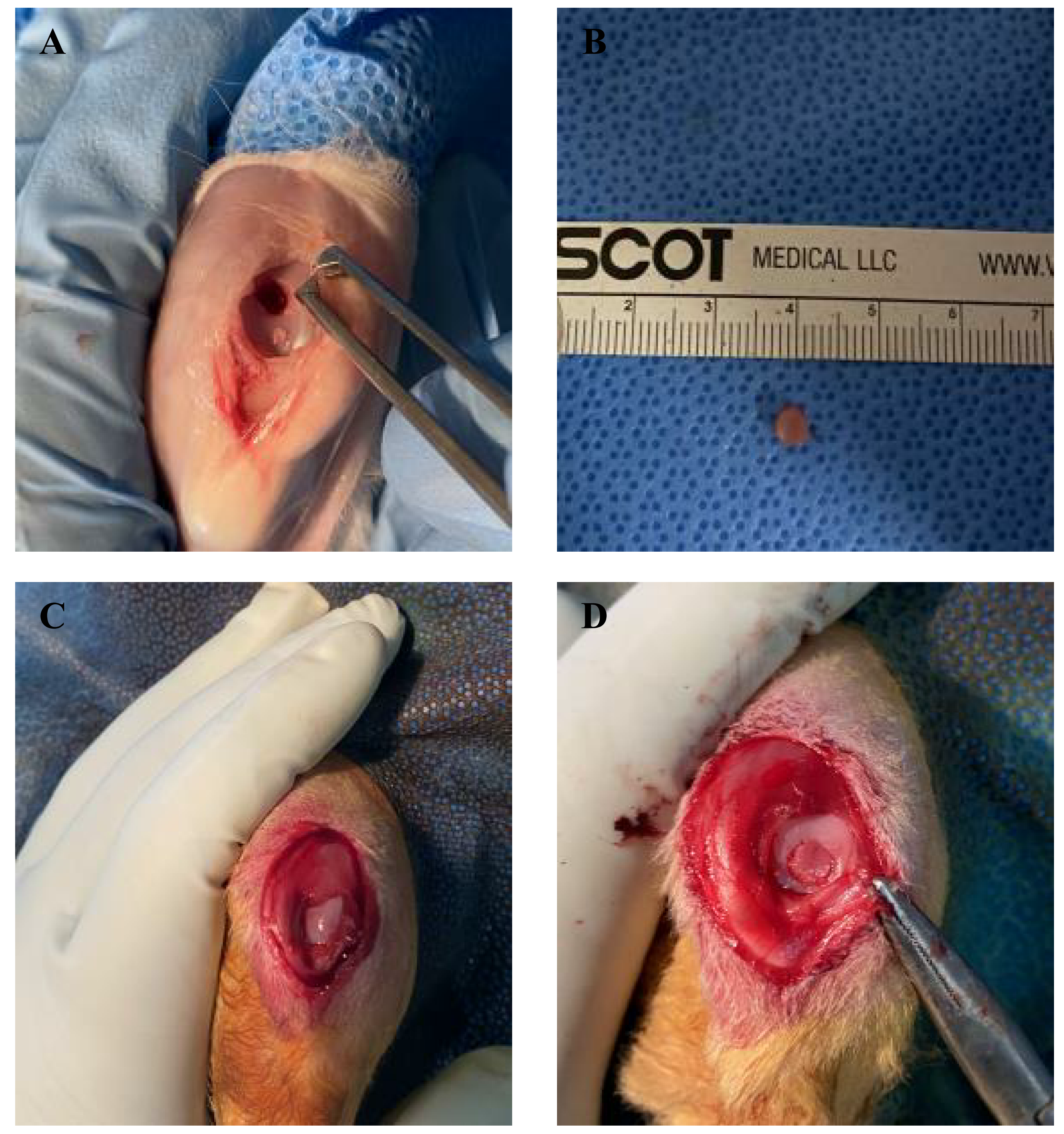

Supplement: S1 Fig — (A) Graft harvested from a donor rabbit knee trochlea. (B) The size of the graft is 4 mm in diameter and 2.5 mm in depth. (C) Fully exposed recipient rabbit knee pulley. (D) Finally, the picture after implantation of the osteochondral graft. (TIF) [file pone.0285733.s001.tif]
